# Deciphering the spectrum of cutaneous lymphomas expressing TFH markers

**DOI:** 10.1038/s41598-023-33031-3

**Published:** 2023-04-20

**Authors:** Marie Donzel, Alexis Trecourt, Brigitte Balme, Olivier Harou, Claire Mauduit, Emmanuel Bachy, Hervé Guesquières, Juliette Fontaine, Nicolas Ortonne, Marie Perier-Muzet, Stéphane Dalle, Alexandra Traverse-Glehen

**Affiliations:** 1 Institut de Pathologie multisites, Hospices Civils de Lyon, Hôpital Lyon Sud, 165 Chemin du Grand Revoyet, 69310 Lyon, Pierre-Bénite France; 2 Hospices Civils de Lyon, Cancer Research Center of Lyon, INSERM U1052, CNRS UMR 5286, CLB, UCBL, Université Lyon 1, Lyon, France; 3 Hospices Civils de Lyon, Service d’Hématologie, Hôpital Lyon Sud, Lyon, France; 4 Biological Immunology, APHP Henri Mondor Hospital, Paris Est Creteil University (UPEC) and INSERM U955 Team Ortonne (NFL), Créteil, France; 5 Department of Dermatology, APHP Henri Mondor Hospital, Créteil, France; 6 Hospices Civils de Lyon, Service de Dermatologie, Hôpital Lyon Sud, Lyon, France

**Keywords:** Cancer, Molecular biology

## Abstract

T-follicular helper (TFH) markers are expressed in the microenvironnement of marginal zone B-cell lymphoma (MZL), and in lymphomas arising from TFH-cells, sometimes making the differential diagnosis difficult. In the skin, the “TFH-spectrum” is poorly defined, going from primary cutaneous lymphoproliferative disorder with small/medium CD4+ T-cells (SMLPD) to cutaneous localizations of systemic angioimmunoblastic T-cell lymphoma (cAITL), and may pass through intermediate forms (primary cutaneous T-follicular helper derived lymphoma, not otherwise specified (PCTFHL,NOS)). We retrospectively analyzed 20 MZL, 13 SMLPD, 5 PCTFHL, and 11 cAITL clinically, histologically, and molecularly, to define tools to differentiate them. Characteristics that might favor the diagnosis of MZL over SMLPD are: multiple skin nodules (p < 0.001), nodular architecture (p < 0.01), residual germinal centers with follicular dendritic cell network (p < 0.001), monotypic plasma cells (p < 0.001), and few staining with PD1 (p = 0.016) or CXCL13 (p = 0.03). PCTFHL and cAITL presented as multiple (p < 0.01) lesions, in older patients (p < 0.01), with systemic symptoms and/or biological alterations (p < 0.01). Immunophenotypic loss of T-cell markers (p < 0.001), BCL6 (p = 0.023) and/or CD10 staining (p = 0.08), and a higher proliferative index (≥ 30%, p = 0.039) favoured these diagnoses over SMLPD. Pathogenic variants were observed by genomic sequencing in 47% of MZL (*TNFAIP3* (32%), *EP300* (21%), *NOTCH2* (16%), *KMT2D* (16%), *CARD11* (10.5%)), 8% of SMLPD (*TET2*), 40% of PCTFHL (*SOCS1* (20%), *ARID1A* (20%)) and 64% of cAITL (*TET2* (63.6%), *RHOA* (36.4%), *NOTCH1* (9%)). This study characterizes the various clinical and histological features between cutaneous lymphomas expressing TFH markers and highlights the value of the interest of screening for genomic mutations in difficult cases.

## Introduction

Since their arrival in the panel of immunohistochemistry, T-follicular helper (TFH) markers have helped improving diagnostic accuracy in many subtypes of lymphomas. Their expression is mainly observed in T-cell lymphomas arising from TFH-cells. In the skin, lymphoma deriving from TFH-cells are poorly defined. Some authors suggest that there is probably a spectrum of different lymphoproliferative diseases arising from TFH cells, with different clinical presentations and prognosis. Primary cutaneous lymphoproliferative disorder with small/medium CD4+ T-cells (SMLPD)^1^ is described, as the beginning of this spectrum, as an asymptomatic and indolent entity^2^. On the other end of the spectrum, cutaneous localizations of angioimmunoblastic T lymphoma^3^ (cAITL) are generally associated with generalized lymphadenopathy and systemic symptoms. Halfway, some authors claim the existence of an emerging entity^4–6^ called primary cutaneous T-follicular helper derived lymphoma, not otherwise specified (PCTFHL, NOS). The clinical presentation of PCTFHL is intermediate between SMLPD and AITL: unique or multiple plaques/nodules with lymphadenopathies or biological alterations (increase LDH, cytopenia, eosinophilia)^6^. The presence of multiple lesions have been associated with an unfavorable clinical course^7^. It seems crucial to find histological or molecular tools to better differentiate those entities which present a distinct aggressiveness and evolution. Furthermore, molecular alterations of PCTFHL are unknown.

TFH-cells are also find in the microenvironnement of B-cell lymphomas, especially marginal zone lymphomas (MZL)^6,8–10^. The clinicopathological characteristics of cutaneous MZL are close to some cutaneous T-cell lymphomas with a TFH phenotype, in particular primary cutaneous CD4+ small/medium T-cell lymphoproliferative disorder (SMLPD)^10^. The distinction between MZL and SMLPD is sometimes difficult and clonality study is not always discriminatory due to cases without monoclonal rearrangement or with both B (BCR) and T-cell receptor (TCR) rearrangements^7^.

Targeted Next Generation Sequencing (TNGS) has proven its interest in daily practice^11^, but few studies have analyzed the molecular profile of cutaneous lymphomas. Concerning AITL, most studies focus on nodal locations, in which mutations of *TET2* (52–76%), *IDH2* (20–45%), *DNMT3A* (30–40%) and *RHOA G17V* (28–70%) are typical^11–16^. The only study currently published in cutaneous localizations of AITL is the one of Leclaire Alirkilicarslan et al., which included 41 patients and found *IDH2 R172K/S* and *RHOA G17V* mutations in 19% and 78% of cases respectively^17^. A recent study also showed a mutation of *DNMT3A*^18^ in one case of SMLPD.

We retrospectively described a series of 49 cutaneous lymphomas with TFH-markers expression, including cases with TFH expression in tumoral cells and cases with TFH hyperplasia in the microenvironnement, to characterize them at a clinicopathological and molecular level and highlight tools to differentiate them.

## Methods

### Patient selection

TFH-cells arising lymphomas (SMLPD and cutaneous localizations of systemic AITL) and cutaneous MZL diagnosed from March 2017 to March 2019 in agreement to the 2016 WHO classification of hematologic malignancies were retrieved from the pathological department of the Lyon Sud University Hospital, France. Intermediate forms between SMLPD and AITL, so-called PCTFHL, were also retrieved.

Inclusion criteria for MZL, PCTFHL and SMLPD were the presence of a monoclonal B- or T-cell population, to limit the inclusion of reactive lymphoid hyperplasia, and enough FFPE material available. All cases had to have benefited from an extensive assessment including either a CT-scan or chest X-ray and abdominal and pelvic ultrasound, to exclude systemic lymphoma with secondary involvement of the skin. MF and/or Sezary syndrome were ruled out by clinicopathological correlation and expert opinion from the French cutaneous lymphomas study group (GFELC). Concerning cutaneous localizations of AITL, cases were included only if they had a known history of systemic AITL, proved by a lymph node biopsy.

### Clinical, biological, and radiological data

Clinical data were retrospectively collected. It included: age at diagnosis, sex, medical history, clinical presentation (number of lesions, localization, systemic symptoms, and lymphadenopathy), biological data (blood count, borreliosis serology), imaging data, received treatments and evolution. Patients were divided into two groups: (i) “indolent” (spontaneous disappearance and/or no relapse after local treatment); or (ii) “refractory/relapsing” (relapse/persistence after initial local treatment requiring chemotherapy or radiotherapy).

### Histological review

Experts pathologists of the French cutaneous lymphomas study group (GFELC)^18–20^ (BB, OH) or of the Lymphopath Network^21^ (ATG, MD) reviewed cases. They recorded: localization (superficial, mid or deep-dermis, hypodermis), architecture (lichenoid (sub-epidermal band), nodular, diffuse, or interstitial (scattered cells in the dermis) pattern), size of cells, epidermotropism (in these cases, results of the blood immunophenotyping were retrieved to exclude cases with a doubtful diagnosis with Sezary syndrome), germinal centers, and associated cells.

### Immunochemistry study

Immunohistochemistry for CD2, CD3, CD4, CD5, CD7, CD8, TFH markers, CD20, CD21, CD23, CD30, CD138, kappa, lambda, and Ki67/Mib1 was performed using standard procedures (Leica BOND-MAX, Leica Microsystems SA, Nanterre, France). In situ hybridization for Epstein-Barr virus (EBV) encoded RNAs (EBER) was performed using a Ventana BenchMark XT automated immunostainer. Protocol and antibodies are detailed in Suppl. Methods and Suppl. Table S1. The proportion of B and T-cells was estimated as the ratio of cells to the total lymphocytic infiltrate. The expression of CD138 was scored semi quantitatively on the basis of the proportion of positive cells in the whole tissue: 0: no staining, 1+: < 10%, 2+: 10 to 25%, 3+: ≥ 25%. Immunochemistry of TFH markers was performed using the following antibodies: PD1 (NAT105; Roche Diagnostics; Meylan, France), CXCL13 (BCA-1; R&D System, Minneapolis, USA), BCL6 (GI191E/A8; Roche Diagnostics; Meylan; France), CD10 (SP67; Roche Diagnostics; Meylan; France), and ICOS (AB105227; Abcam, Cambridge, UK). Their expression was scored quantitatively on the basis of the proportion of positive cells in the whole T-cells infiltrate. They are presented with their standard deviation. The proliferative index was evaluated as the percentage of Ki67-positive cells relative to the total lymphocyte population.

### Clonality study and targeted next generation sequencing (TNGS)

Clonality study was performed according to the EuroClonality/BIOMED-2 protocol^22^. IGH and IGK assays were used for BCR study, with the following primers: FR1, FR2, FR3, DH–JH, Ig kappa, and Ig lambda. TCR Clonality was assessed using the BIOMED-2 primer sets for TCR gamma (TCRG). TNGS have been performed as previously reported^11,23^. The applied panel of 47 genes is detailed in Suppl. Table S2. Only pathogenic variants with a minimal depth of 700× were retained. Supplemental quality data are available in Suppl. Table S4.

### Statistical analysis

Statistical analysis has been performed using the Medistica pvalue.io software^24^, performing univariate analysis for descriptive variables. Kruskal–Wallis test was used to study ordinal qualitative ordinal variables (immunohistochemistry results) and quantitative variables (age) in the 4 independent groups (CMZL, SMLPD, PCTFHL, and cAITL). Fisher’s exact test or χ^2^ test were used to study nominal qualitative variables (clinical and histological characteristics) in the different groups.

### Compliance with ethical standards in research

This study was performed in accordance with the Declaration of Helsinki. Informed consent was obtained from all subjects and/or their legal guardian(s) according to the guidelines of the French Bioethics Law. The protocol has received the validation of the local ethics committee (Ethics committee of the Hospices Civils de Lyon, number 21_5588).

## Results

Sixty-seven cases corresponding to 27 MZL, 21 SMLPD, 8 PCTFHL and 11 cAITL were retrieved. Seven MZL were excluded due to the presence of a systemic MZL. Eight SMLPD were excluded: 3 because the material was exhausted, 4 due to the absence of any monoclonal T-cell population, and one because further evaluation led to reclassification as MF. Three PCTFHL were excluded, two due to the absence of TCR clonal rearrangement, and one due to the presence of Sezary cells in the blood, leading to reclassification as Sezary syndrome. Twenty MZL, 13 SMLPD, 5 PCTFHL, and 11 cAITL were finally included.

### Clinical presentation (Table 1)

**Table 1:** Clinical data of patients with MZL, SMLPD, PCTFHL, and cutaneous location of AITL including clinical presentation, treatment, and evolution.

			MZL (n, %)	SMLPD (n, %)	p (MZL vs. SMLPD)	PCTFHL (n, %)	cAITL (n, %)	p (all subtypes)
Clinical characteristics	Number of cases		20	13		5	11	
	Mean age (range)		56 (27–84)	53 (25–78)	0.66	74 (47–91)	74 (56–92)	** < 0.01**
	Sex ratio		0.5	0.4	0.52	1	0.8	**0.032**
	Unique lesion		9 (45)	13 (100)	** < 0.01**	0 (0)	0 (0)	** < 0.001**
	≥ 2 lesions		11 (55)	0 (0)	** < 0.001**	5 (100)	11 (100)	** < 0.001**
	Positive borrelia serology		2 (10)	0 (0)	1	0 (0)	0 (0)	0.84
	Clinical presentation	Erythematous nodule(s) or papule(s)	16 (80)	9 (69)	0.68	3 (60)	2 (18)	** < 0.01**
		Erythematous and scaly patch	4 (20)	4 (31)	0.68	2 (40)	3 (27)	0.77
		Maculo-papular eruption	0 (0)	0 (0)	NA	0 (0)	6 (55)	** < 0.001**
	Location of lesions	Upper limbs	17 (40)	2 (15)	** < 0.001**	NA		NA
		Head and neck	3 (10)	4 (30)	0.39			
		Trunk, abdomen	17(40)	7 (55)	0.11			
		Lower limbs	5 (10)	0 (0)	0.13			
	Erythrodermia		0 (0)	0 (0)	1	1 (20)	2 (18)	**0.048**
	Systemic symptoms		0 (0)	0 (0)	1	0 (0)	11 (100)	** < 0.001**
	Biological alterations		0 (0)	0 (0)	1	2 (40)	10 (91)	** < 0.001**
	Treatment	None/spontaneous disappearance	0 (0)	1 (8)	0.39	0 (0)	0 (0)	0.59
		Topical corticosteroids	11 (55)	3 (23)	0.07	5 (100)	6 (55)	**0.027**
		Doxycycline	10 (50)	3 (23)	0.12	0 (0)	0 (0)	** < 0.01**
		Surgery	15 (75)	10 (77)	1	0 (0)	1 (9)	** < 0.001**
		Radiotherapy	3 (15)	0 (0)	0.26	1 (20)	0 (0)	0.24
		Chemotherapy	0 (0)	0 (0)	1	2 (40)	10 (91)	** < 0.001**
	Evolution	Disappearance of lesions	11 (55)	11 (84)	0.13	1 (20)	1 (9)	** < 0.001**
		Relapse after DC or surgery	9 (45)	2 (15)	0.13	3 (60)	10 (91)	** < 0.01**
		High grade lymphoma requiring chemotherapy	0 (0)	0 (0)	1	0 (0)	10 (91)	** < 0.001**
		Lost	0 (0)	0 (0)	1	1 (20)	0 (0)	0.1

Locations of lesions are the total number of lesions in the location section is higher than the number of patients, as some patients had multiple lesions in different locations.

NA: not analyzed, MZL: marginal zone lymphoma, SMLPD: primary cutaneous (CD4+) small/medium T-cell lymphoproliferative disorder; PCTFHL: primary cutaneous T-follicular helper derived lymphoma; cAITL: cutaneous localization of angio-immunoblastic T lymphoma.

Significant values are in bold.

*MZL* presented as erythematous papules or nodules (16/20, 80%) or erythematous-squamous plaque (4/20, 20%). Lesions were more often multiple (11/20, 55%) with, on average, 3 lesions per patient [range: 2–5]. Median age was of 56-years-old [27–84]. Locations were upper limbs (40%), trunk and abdomen (40%), lower limbs (10%), and head and neck (10%). Borrelia serology was positive in 2 cases. None had lymphadenopathy. Blood counts were normal.

*SMLPD* presented as unique (100%) erythematous nodule (9/13, 69%) or erythematous-squamous plaque (4/13, 31%), mainly located on the trunk (55%) or head and neck (30%), with a median age of 56-years-old [25–78]. Borrelia serology was negative in all cases. Blood counts were normal.

*PCTFHL* presented as multiples (5/5, 100%) erythematous-squamous plaques (2/5, 40%) or erythematous nodules (2/5, 40%), or papules (1/5, 20%). One case (PCTFHL no 5) presented a monoclonal circulating CD4+ T-cell population without immunophenotyping feature of Sezary syndrome. The median age was 74-years-old [47–91]. Two PCTFHL presented biological alterations (anemia: Haemoglobin 11.2 g/dl and increased LDH level: 352 U/L).

Most *cAITL* presented as a maculopapular rash (6/11, 55%), with a median age of 74-years-old [56–92]. Other cases presented as multiple (100%) erythematous papules (2/11, 18%) or erythematous-squamous plaques (3/11, 27%). In AITL no 11 the disease appeared as a unique, infiltrated, necrotic plaque of the abdomen. In six cases, biopsies were performed at the onset of the disease (at diagnosis). For the five other cases, cutaneous lesions occurred during a relapse of the disease (after a first line of chemotherapy). All patients described systemic symptoms (deterioration in the general condition: n = 11/11, pruritus: n = 3/11, fever: n = 4/11) or biological alterations (bi/pancytopenia: n = 7/11, increase in LDH [338–600]: n = 4/11).

### Histological review and immunohistochemistry study (Table 2)

**Table 2:** (A) Histological and immunohistochemical characteristics of patients with MZL, SMLPD, PCTFHL, and cutaneous location of AITL. Average of expression of TFH markers are associated with their respective ranges of expression. (B) Details of immunohistochemistry results illustrating the average of stained cells and their respective ranges of expression.

(A)		MZL (n, %)	SMLPD (n, %)	p (MZL vs. SMLPD)	PCTFHL (n, %)	cAITL (n, %)	p (among TFH spectrum)
Total		20	13		5	11	
Hypodermis involvement		7 (35)	6 (46)	0.52	1 (20)	0 (0)	**0.029**
Architecture	Nodular	19 (95)	7 (54)	** < 0.01**	1 (20)	2 (18)	0.17
	Diffuse	0 (0)	2 (15)	0.15	0 (0)	0 (0)	0.65
	Interstitial	0 (0)	0 (0)	1	1 (20)	9 (82)	** < 0.001**
	Lichenoid (sub epidermal band)	1 (5)	4 (31)	0.06	3 (60)	0 (0)	**0.023**
Epidermotropism		0 (0)	0 (0)	1	1 (20)	0 (0)	0.17
Spongiosis		0 (0)	5 (38)	** < 0.01**	0 (0)	0 (0)	**0.027**
Residual germinal centers		12 (60)	0 (0)	** < 0.001**	0 (0)	0 (0)	1
Follicular dendritic cell network		15 (75)	0 (0)	** < 0.001**	0 (0)	0 (0)	1
Presence of eosinophils		4 (20)	6 (46)	0.14	3 (60)	7 (64)	0.71
Plasma cells	Presence of plasma cells	19 (95)	12 (92)	1	2 (40)	4 (36)	** < 0.01**
	< 10% of the infiltrate	7 (35)	11 (85)	**0.019**	2 (100)	4 (100)	**0.039**
	10–24%	6 (30)	1 (8)	0.2	0 (0)	0 (0)	1
	≥ 25% of the infiltrate	7 (35)	0 (0)	**0.027**	0 (0)	0 (0)	1
	Monotypia	16 (75)	0 (0)	** < 0.001**	0 (0)	0 (0)	1
	Kappa	5 (25)	0 (0)	0.13	0 (0)	0 (0)	1
	Lambda	11 (55)	0 (0)	** < 0.01**	0 (0)	0 (0)	1
Loss of T cell markers		0 (0)	0 (0)	1	4 (80)	5 (45.5)	** < 0.001**
TFH markers (number of positive cases)	PD1	18 (90)	13 (100)	1	5 (100)	11 (100)	1
	CXCL13	15 (75)	13 (100)	0.13	5 (100)	11 (100)	1
	BCL6	5 (25)	6 (46)	0.27	5 (100)	11 (100)	< 0.01
	CD10	0 (0)	0 (0)	1	2 (40)	3 (28)	0.067
	ICOS	6 (30)	4 (31)	1	NA	NA	NA
Monoclonal B cell population		20 (100)	2 (15)	** < 0.001**	0 (0)	2 (18)	1
Monoclonal T cell population		1 (5)	13 (100)	** < 0.001**	5 (100)	7 (64)	**0.024**

(B)		MZL (%, [range])	SMLPD (%, [range])	p (MZL vs. SMLPD)	TFHL (%, [range])	cAITL (%, [range])	p (among TFH spectrum)
CD20+ B cells		55 [25–75]	35 [15–50]	** < 0.01**	20 [0–50]	5 [0–10]	** < 0.001**
T cells	CD3+ T cells	43.5 [25–75]	61.5 [50–80]	** < 0.001**	80 [50–100]	95 [90–100]	** < 0.001**
	CD4+ T cells	75 [50–90]	75 [50–85]	0.51	70 [60–75]	70 [60–90]	0.69
	CD8+ T cells	25 [10–40]	25 [20–40]	0.51	30 [25–40]	30 [10–40]	0.15
TFH markers (average of stained cells, [range])	PD1	19 [0–80]	49 [10–90)	**0.016**	71 [10–75]	40.5 [10–75]	0.12
	CXCL13	8 [025]	20 [1075)	**0.03**	30 [10–50]	16.5 [5–70]	0.79
	BCL6	2.5 [0–10]	11 [0–75)	0.27	20 [10–40]	21 [10–70]	**0.024**
	CD10	0	0	1	5 [0–10]	4 [0–20]	0.083
	ICOS	1 [0–1]	1 [0–1)	1	NA	NA	NA
KI67/Mib1		18 [5–30]	15 [5–30)	0.094	32 [10–80]	35 [10–70]	**0.039**

BCL6: B-cell lymphoma 6 protein; CD: Cluster of differentiation; CXCL13: C-X-C chemokine receptor type 5; NA: Not analyzed; PD1: Programmed cell death 1; MZL: marginal zone lymphoma, NA: statistical analysis not performed; SMLPD: (Primary cutaneous CD4+) small/medium T-cell lymphoproliferative disorder; PCTFHL: primary cutaneous T-follicular helper derived lymphoma; cAITL: cutaneous localization of angio-immunoblastic T lymphoma.

Significant values are in bold.

**Figure 1:**
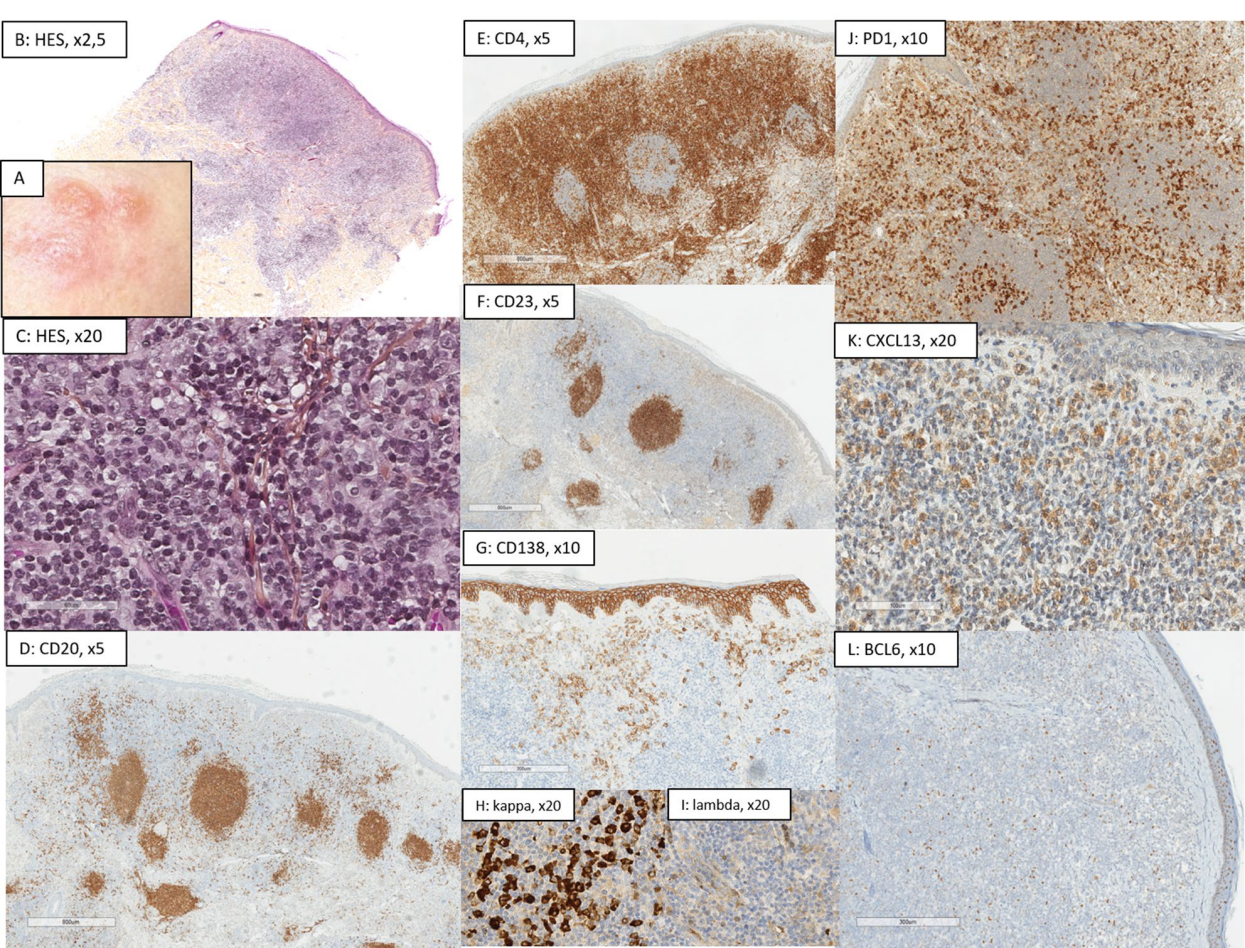
Histological presentation of a primary cutaneous marginal zone lymphoma (MZL, case no 6). (**A**) Clinical presentation as 3 erythematous nodules of the face; (**B**) (Hematoxylin–Eosin-Saffron (HES), ×2.5): Nodular and diffuse architecture involving all the dermis; (**C**) (HES, ×20): small hyperchromatic cells associated with histiocytes; (**D**) (CD20, ×20) and (**E**) (CD4, ×20): Association of B and T-cells in equal proportions (among 50% of each); (**F**) (CD23, ×10): Residual germinal centers, underlined by a network of CD23+ follicular dendritic cells; (**G**–**I**) (CD138 × 10, kappa/lambda, ×20); Association to numerous monotypic kappa plasma cells located outside of the B-cell nodules; (**J**) (PD1, ×10), (**K**) (CXCL13, ×20), and (**L**) (BCL6, ×10): small T-cells of the background harbor a TFH phenotype (PD1 30%, CXCL13 15%, BCL6 10%, CD10 negative). Proliferative index using Ki67/Mib1 was evaluated a 20% (picture not shown). The diagnosis of MZL was confirmed thanks to the presence of a monoclonal B-cell population. This case displayed no pathogenic variant in TNGS.

**Figure 2:**
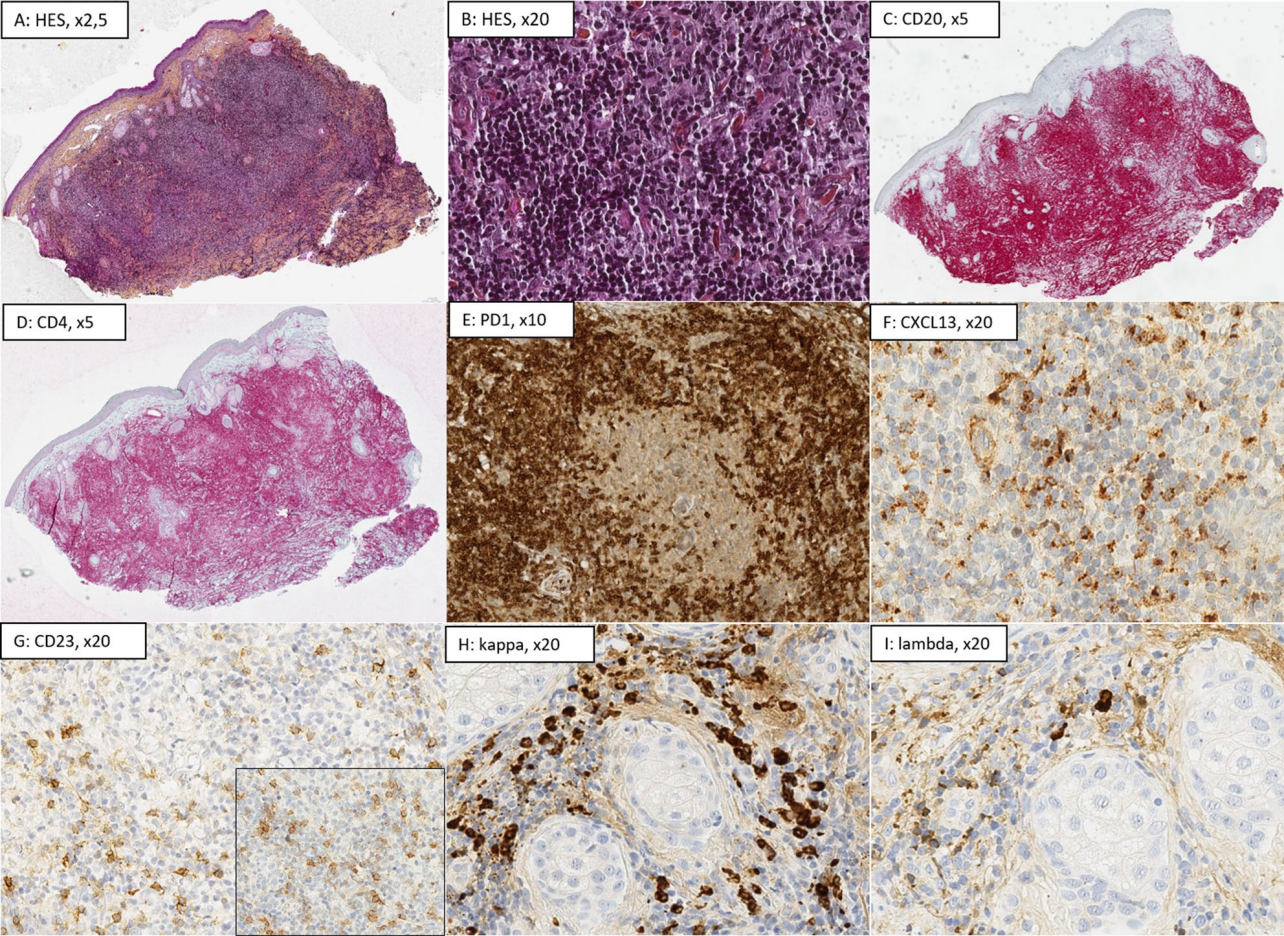
Histological presentation of a primary cutaneous marginal zone lymphoma (MZL, case no 2) with significant TFH hyperplasia, illustrating the diagnostic difficulty between SMLPD and MZL. (**A**) (Hematoxylin–Eosin-Saffron (HES), ×10): Nodular and diffuse architecture involving all the dermis, well separated from the epidermis by a “grenz-zone”; (**B**) (HES, ×20): Small hyperchromatic cells; (**C**) (CD20, ×5) and (**D**) (CD4, × 5): The infiltrate is composed of admixed B-cells (50%) and CD4+ T-cells (50%) ; (**E**) (PD1, ×10) and (**F**) (CXCL13, ×20): Strong expression of TFH markers in the microenvironnement (PD1: 80%, CXCL13: 25%); (**G**) (CD23, ×20): Absence of residual CD23+ follicular dendritic cells network; (**H**,**I**) (kappa and lambda, ×20); Plasma cells were few (< 10%) but presented a kappa monotypia. The diagnosis of MZL was confirmed thanks to molecular data; the presence of a monoclonal B-cell population, and pathogenic variants of ITPKB, NOTCH2, TNFAIP3, and KMT2D genes using TNGS.

*MZL* (Figs. 1, 2) presented a nodular architecture (19/20, 95%), with residual germinal centers (GC) (12/20, 60%) and a CD23+ follicular dendritic cell (FDC) network (15/20, 75%). One case presented a lichenoid architecture. Hypodermis involvement was obseved in 7/20 cases (35%). MZL were composed of more B (55% (± 17.2)) than T-cells (43.5% (± 16.1)), but 4 had more T-cells than B-cells, and 2 had equal proportions of both. CD138+ plasma cells were present in almost all cases (n = 19/20, 95%), representing more than 10% of cells in 65% of cases, and being monotypic in 75% of cases. Eosinophils were present in 4/20 cases (20%). TFH markers were not constantly expressed in MZL cases; PD1: n = 18/20; CXCL13: n = 15/20; BCL6: n = 5/20; no CD10 expression, and average of stained were low; PD1: 19% (± 17.6); CXCL13: 8% (± 8.01); BCL6: 2.5% (± 4.44). ICOS was expressed in 6 cases (< 1% of stained cells). When positive, PD1 was expressed with a high intensity on the T-cells of the germinal centers, and, with a weak or moderate staining in small T-cells of the background. Ki67 was evaluated at 18% (± 6.1).

**Figure 3:**
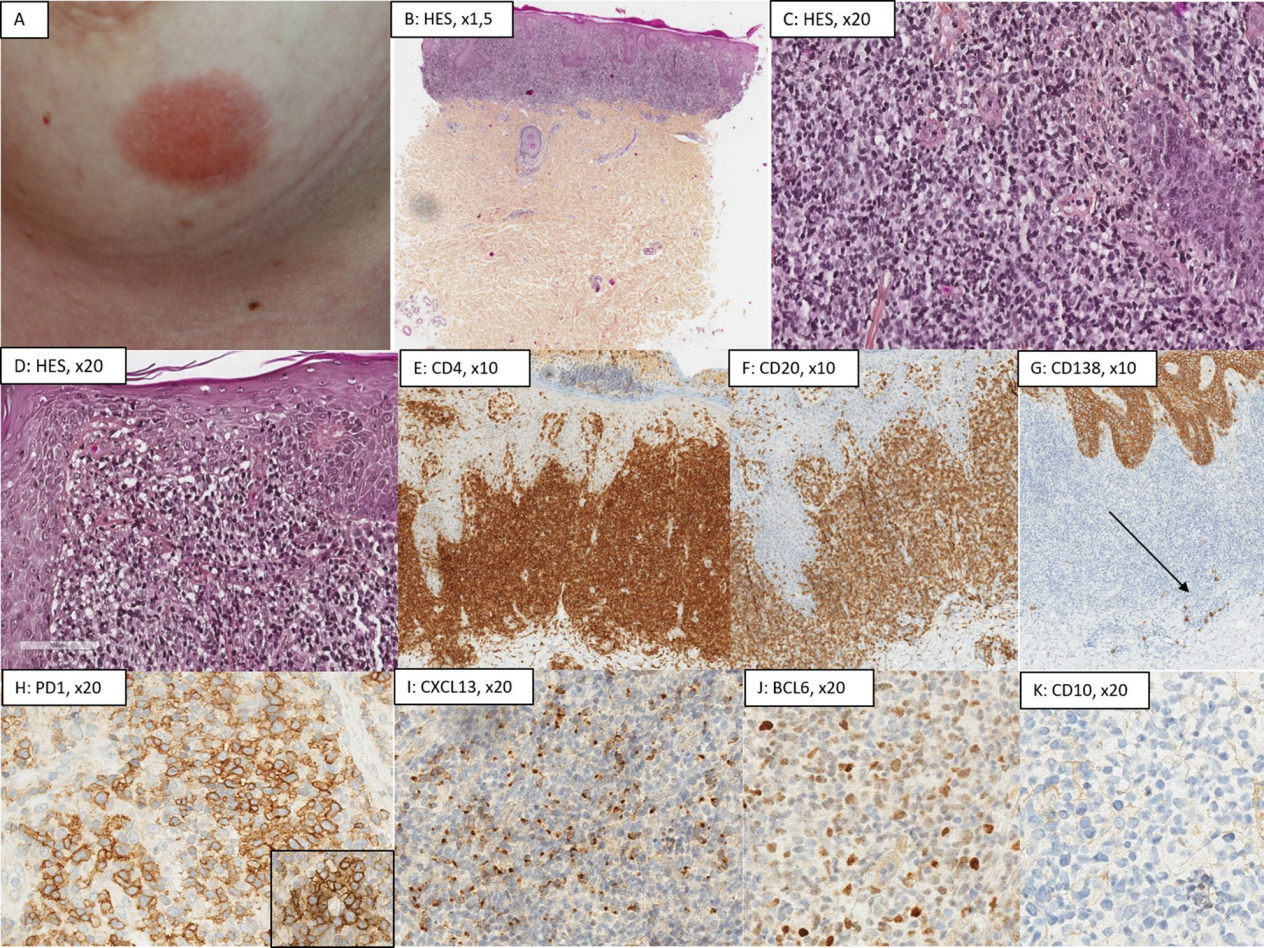
Histological presentation of a primary cutaneous CD4+ small/medium T-cell lymphoproliferative disorder (SMLPD, case no 7). (**A**) Clinical presentation as an infiltrated erythematous-squamous plaque of the left breast; (**B**) (Hematoxylin–Eosin-Saffron (HES), ×1.5), (**C**,**D**) (HES, ×20): lichenoid pattern constituted of atypical cells, small-medium sized; (**E**) (CD4, ×10) and (**F**) (CD20, ×10): slight predominance of T-cells (60%), with a CD2+ CD5+ CD7+ phenotype, and a significant predominance of CD4+ T-cells (75%) instead of CD8+ T-cells (25%). (**G**) (CD138, ×10): very few associated plasma cells (black arrow), which were polytypic for kappa and lambda, and located outside of the proliferation in this particular case, but scattered among T-cells in most SMLPD cases; (**H**) (PD1, ×20), (**I**) (CXCL13, ×20), (**J**) (BCL6, ×20) and (**K**) (CD10, ×20): huge expression of TFH markers except for CD10 which was negative (PD1 90%, CXCL13 40%, BCL6 25%). As illustrated, PD1 expression shows a higher intensity in medium/large cells, and some PD1+ cells also tended to form “rosettes” around large lymphocytes (insert). Proliferative index using Ki67/Mib1 was evaluated a 15%, and no follicular dendritic cells network (pictures not shown). The diagnosis of SMLPD was confirmed thanks to molecular data; the presence of a monoclonal T-cell population. This case displayed an isolated TET2 pathogenic variant using TNGS.

*SMLPD* (Fig. 3) presented a nodular (7/13, 54%), diffuse (2/13, 15%), or lichenoid (4/13, 31%) pattern, without residual GC nor FDC network, and with hypodermis involvement in 6/13 cases (46%). Eosinophils were present in 6/13 cases (46%). Infiltrate was composed of a majority of T (61.5% (± 11.4)) rather than B-cells (35% (± 12)), except in 4 cases in which T and B-cells were equally distributed. There was no loss of T-cell markers. Plasma cells were present (n = 12/13, 92%), but represented less than 10% of cells in most cases (n = 11/12, 85%) and were always polytypic. PD1 and CXCL13 were seen in all cases, BCL6 in 6 cases and ICOS in 4 cases. Average of stained T-cells were; PD1: 49% (± 33.7), CXCL13: 20% (± 20.0), BCL6: 11% (± 20.7), ICOS: < 1%. There was no CD10 expression. PD1 showed a higher intensity in medium/large cells, and some PD1+ cells tended to form clusters or “rosettes” around large lymphocytes (Fig. 3H). Ki67 was evaluated at 15% (± 6.6).

**Figure 4:**
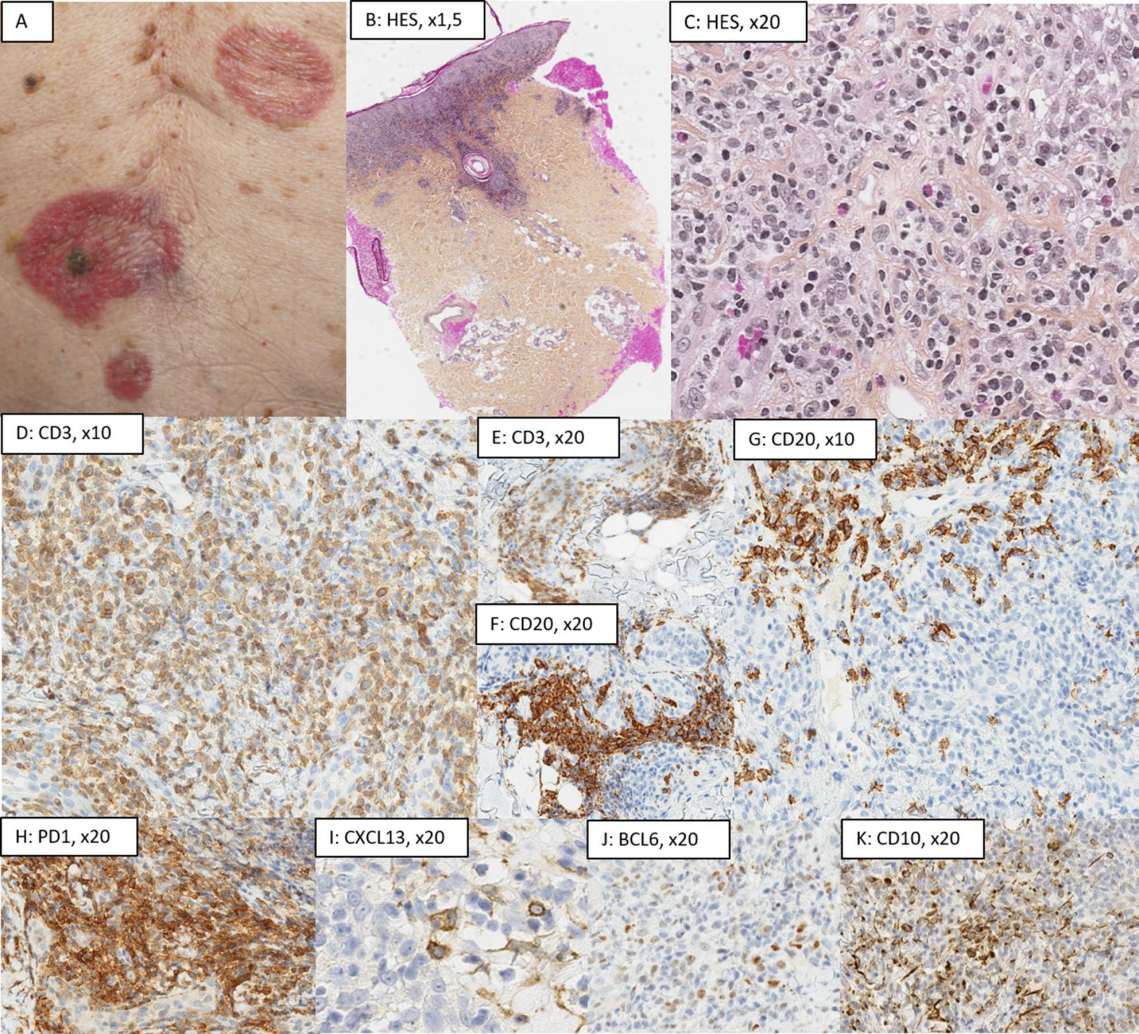
Histological presentation of a primary cutaneous T-follicular helper lymphoma (PCTFHL, case no 3). (**A**) clinical presentation as pruriginous annular, erythematous and infiltrated lesions, located in the back, the right preauricular region, and the chin; (**B**) (Hematoxylin–Eosin-Saffron (HES), ×1.5) and (**C**) (HES, ×20): lichenoid pattern with epidermotropism. Infiltrate was constituted of atypical cells, small-medium sized, associated with eosinophilic polynuclear (black arrow); (**D**,**E**) (CD3, ×10, ×20) and (F,G (CD20, ×10, ×20): slight predominance of T-cells (60%), with a partial loss of CD7, associated to numerous B-cells (without CD30 expression or RNA EBER positivity); (**H**) (PD1, ×20), (**I**) (CXCL13, ×20), (**J**) (BCL6, ×20) and (**K**) (CD10, ×20): huge expression of TFH markers (PD1 80%, CXCL13 50%, BCL6 20% CD10 10%). Proliferative index using Ki67/Mib1 was evaluated a 35% (picture not shown). The diagnosis of PCTFHL was confirmed thanks to molecular data; the presence of a monoclonal T-cell population. This case displayed a pathogenic variant of SOCS1 using TNGS.

In *PCTFHL* (Fig. 4), architecture was lichenoid (3/5, 60%), interstitial (1/5, 20%) or nodular (1/5, 20%). There were no epidermotropism, excepted in PCTFHL no 3. Hypodermis involvement was rare (1/5, 20%). Eosinophils were seen in 3 PCTFHL (60%). There were no residual GC nor FDC network. Plasma cells were rare (n = 2/5, 40), representing less than 10% of the cells without monotypia. T-cell markers were lost in 80% of PCTFHL (CD7: 3/5, CD5: 1/5). PD1, CXCL13 and BCL6 were expressed in all cases, and CD10 expression was observed in 2/5 PCTFHL. Average of stained T-cells in PCTFHL were; PD1: 71% (± 21.3); CXCL13: 30% (± 20.0); BCL6: 20% (± 12.2), and CD10: 4% (± 5.48). ICOS had not been performed. PD1 was expressed with a moderate to high intensity on the T-cells, with a diffuse pattern. Ki67 was evaluated at 32% (± 27.7).

**Figure 5:**
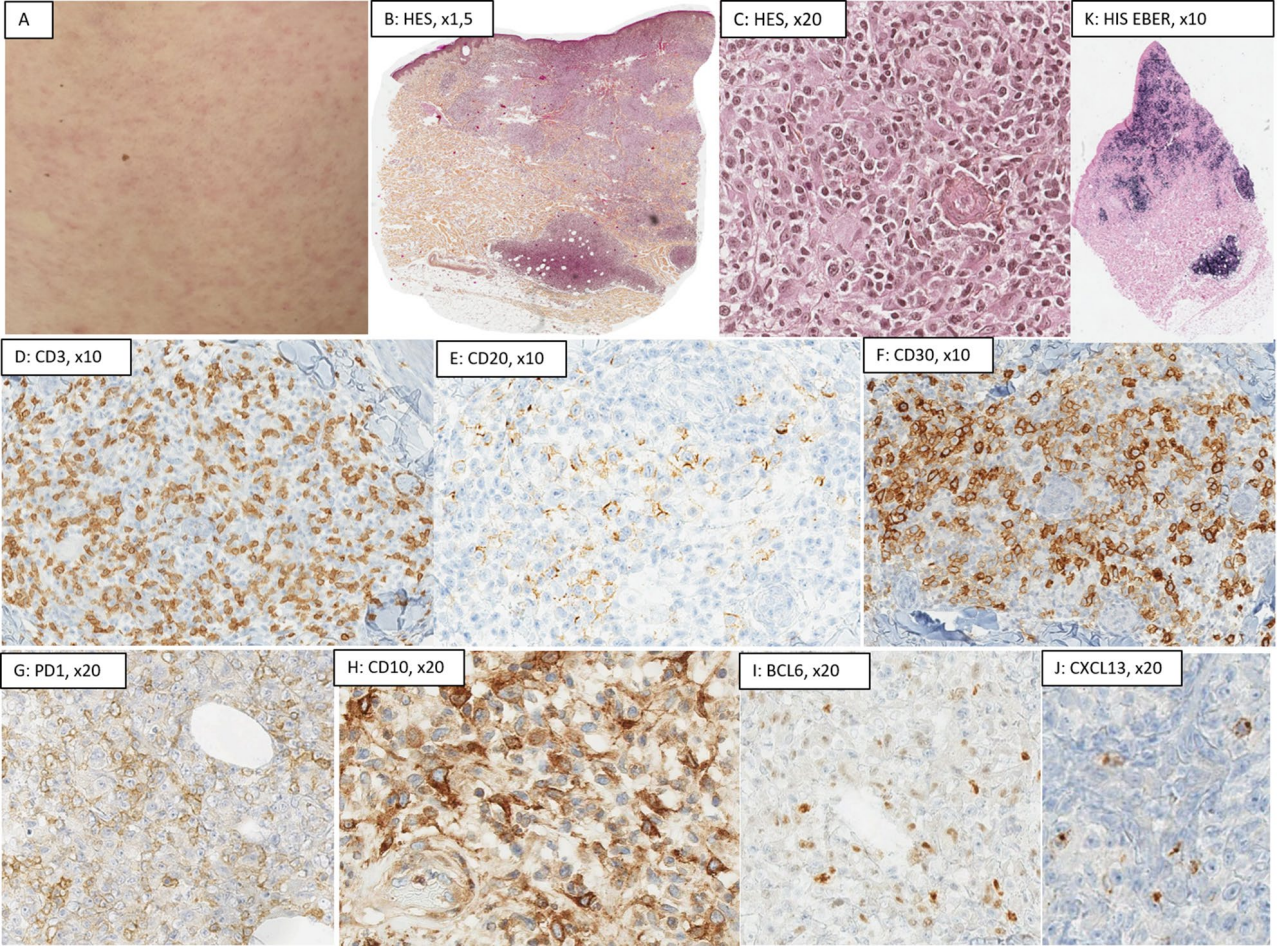
Histological presentation of a cutaneous localization of angioimmunoblastic T-cell lymphoma (cAITL, case no 2). (**A**) Clinical presentation as maculopapular rash reaching 50% of the skin surface; (**B**) (Hematoxylin–Eosin-Saffron (HES), ×1.5) and (**C**) (HES, ×20): nodular and diffuse architecture, constituted of atypical cells, small-medium sized, associated to large immunoblastic cells; (**D**) (CD3, ×20): Atypical cells presented a T-cell phenotype, with loss of the CD7; (**E**) (CD20, ×10) and (**F**) (CD30, ×10): large immunoblastic B-cells, which intensely expressed CD30. (**K**) (hybridization in situ (HIS) RNA EBER, ×1.5): CD30+ B-cells also presented an overexpression of RNA EBER of the Epstein Barr Virus; (**G**) (PD1, ×20), (**H**) (CD10, ×20), (**I**) (BCL6, ×20) and (**J**) (CXCL13, ×20): Expression of TFH markers (PD1 40%, CD10 25%, BCL6 10%, and CXCL13 10%). Proliferative index using Ki67/Mib1 was evaluated a 70% (picture not shown). The diagnosis of cAITL was confirmed thanks to molecular data; the presence of a monoclonal T-cell population associated with a minor monoclonal B-cell population. This case did not display any pathogenic variant using TNGS.

In *cAITL* (Fig. 5), infiltrates were sparse; architecture was mostly interstitial (9/11, 82%) than nodular (2/11, 18%). Plasma cells were rare (n = 4/11, 36%), representing less than 10% of the cells without monotypia. Eosinophils were seen in 7 cAITL (64%). There were no residual GC nor FDC network. Hypodermis involvement was never seen. CD30+ B-cells were seen in 8/11 cAITL, with overexpression of ARN EBERs using in situ hybridization in 1/11 case. T-cell markers were lost in 45.5% of cAITL (CD7: 4/11, CD5: 1/11). PD1, CXCL13 and BCL6 were expressed in all cases, and CD10 expression was observed in 3/11 cAITL (28%). Average of stained T-cells in cAITL were; PD1: 40.5% (± 19.7); CXCL13: 16.5% (± 16.7); BCL6: 21% (± 17.7), and CD10: 4% (± 6.74). ICOS had not been performed. PD1 was expressed with a moderate to high intensity on the T-cells, with a diffuse pattern. Ki67 was evaluated at 35% (± 22).

### Clinical and histological comparison between CMZL and SMLPD

Both CMZL and SMLPD presented as erythematous nodules (80% vs. 69%, p = 0.68), but lesions were more often multiples in MZL (55% vs. 0%, p < 0.001). Histologically, nodular architecture (95% vs. 54%, p < 0.01), presence of residual germinal centers with FDC network (75% vs. 8%, p < 0.001), and monotypic plasma cells (75% vs. 0%, p < 0.001) were in favor of MZL *versus* SMLPD. Lichenoid architecture was not significantly different in these two subgroups (p = 0.06), nor was the presence of eosinophils (p = 0.14). B-cells were more abundant in MZL (55% vs. 35%, p < 0.01), whereas T-cells were more abundant in SMLPD (61.5% vs. 43.5%, p < 0.001). PD1 expression was lower in MZL compared to SMLPD (19% vs. 49%, p = 0.016). CXCL13 (8% vs. 20%, p = 0.03) and BCL6 (2.5% vs. 11%, p = 0.27) were less expressed in CMZL than SMLPD.

### Comparison between lymphomas of the TFH spectrum

PCTFHL and cAITL arised in older patients (p < 0.01) than SMLPD. Clinically, presentation as maculopapular rash was caracteristical of cAITL (p < 0.001). Presence of multiple lesions (nodules or papules) may guide the diagnosis toward these subtypes rather than SMLPD (p < 0.001), nodules being more characteristics of SMLPD or PCTFHL than cAITL (p < 0.01). Histologically, lichenoid architecture was suggestive of PCTFHL or SMLPD instead of cAITL (p = 0.023), in which interstitial architecture was more frequent (p < 0.001). Eosinophils were more evocative of a lesion of the TFH spectrum (CMZL: 20% vs. SMLPD 46%, PCTFHL 80%, cAITL 64%, p < 0.01) but were not statistically different within the spectrum of TFH lymphomas (p = 0.71). Losses of T-cell markers were seen only in PCTFHL (80%) and cAITL (45%) (< 0.001). PD1 was more often expressed in PCTFHL compared to other subtypes, without significant statistical differences (SMLPD: 49%, PCTFHL: 71%, cAITL: 40.5%, p = 0.12). There were no statistical differences between the expressions of CXCL13 in lymphomas of the TFH spectrum (SMLPD: 20%, PCTFHL: 30%, cAITL: 16.5%, p = 0.79). BCL6 was significantly more frequent in PCTFHL and cAITL than SMLPD (SMLPD: 11%, PCTFHL: 20%, cAITL: 21%, p = 0.024). CD10 was expressed only in PCTFHL and cAITL (p = 0.083). Proliferative index was slightly more elevated in intermediate forms and systemic lymphomas (SMLPD: 15% (± 6.60), PCTFHL: 32% (± 27.7) and cAITL: 35% (± 22.0), p = 0.039).

### Treatments and evolution (Tables 1, 4)

*MZL* treatments consisted of topical corticosteroids (11/20, 55%), doxycycline (10/20, 50%), often associated with surgical excision (15/20, 75%). Nine cases suffered local relapses, treated by topical corticosteroids (6/9), radiotherapy (3/9).

*SMLPD* treatments consisted in topical corticosteroids (betamethasone dipropionate or clobetasol propionate, 3/13, 23%), antifungal cream (1/13, 8%), antibiotic therapy (21 days of doxycycline, 3/13, 23%), alone or associated with surgical excision (10/13, 77%). One case presented a spontaneous disappearance after biopsy. All cases presented an indolent evolution. Among the three cases which did not undergo surgery, two suffered a relapse after dermocorticoids disruption.

Among *PCTFHL*, the median follow-up was of 20 months. The case no 5 with a monoclonal circulating T-cell population responded completely under topical corticosteroids and was considered indolent. Three were refractory/relapsing patients; one received topical corticosteroids with a remitting relapsing course (median follow-up of 50 months), one received radio-chemotherapy (gemcitabine), and the last received methotrexate. The fifth was lost to follow-up.

All *cAITL* were treated by chemotherapy (9 CHOP: cyclophosphamide, doxorubicin, vincristine, and prednisone (CHOP) and 2 GEMOx: gemcitabine, oxaliplatin).

### Clonality study (Tables 2, 3, 4)

With the presence of a monoclonal population being inclusion criteria, BCR rearrangements were present in all MZL and TCR rearrangements in all SMLPD and PCTFHL. The same clone was found in the skin biopsy for the PCTFHL case with a circulating T monoclonal population.

Concerning cAITL, 5 presented a monoclonal T-cell population, 2 presented both BCR and TCR rearrangement, results were undetermined in 3 cases, and one did not display any monoclonal population (AITL no 9).

Two SMLPD and one MZL presented both BCR and TCR rearrangements. The 2 SMLPD presented characteristics typical of SMLPD: unique nodules, 66–80% of T-cells, absence of FDC network, 5–20% polytypic plasma cells, medium PD1/CXCL13 staining (10–30%). The MZL case presented characteristics typical of MZL: multiple lesions, majority of B-cells (60%), nodular pattern, residual germinal centers, 25% of plasma cells with lambda monotypia, low PD1/CXCL13 staining (10%).

### TNGS analysis (Tables 3, 4)

**Table 3:** Results of molecular biology analysis: clonality study and Targeted Next-Generation Sequencing (TNGS) put in parallel with clinical evolution of patients and their received treatments.

No	Follow-up (months)	Form	Received treatments	Monoclonal B-cell population	Monoclonal T-cell population	Pathogenic variants
MZL 1	30	Indolent	TC, DXC	+	−	CARD11, EP300, TNFAIP3
MZL 2	214	Relapse	TC, DXC	+	−	ITPKB, NOTCH2, TNFAIP3, KMT2D
MZL 3	80	Indolent	**SE**	+	−	0
MZL 4	74	Indolent	**SE**	+	−	NA
MZL 5	16	Refractory	**SE,** RT	+	−	CARD11, KMT2D, NOTCH2, TNFAIP3
MZL 6	2	Relapse	TC, DXC, RT	+	−	0
MZL 7	22	Indolent	TC, DXC	+	−	0
MZL 8	66	Indolent	TC, DXC	+	−	TNFAIP3, EP300, PLCG2
MZL 9	9	Relapse	TC, DXC, **SE**	+	−	0
MZL 10	24	Indolent	TC, DXC, **SE**	+	−	CREBBP, EP300
MZL 11	10	Relapse	**SE**, DC	+	−	KMT2D
MZL 12	486	Relapse	TC, DXC, **SE**	+	−	CIITA, EP300, GNA13, NOTCH2, TNFAIP3
MZL 13	65	Relapse	TC, **SE**	+	−	0
MZL 14	56	Indolent	**SE**	+	−	0
MZL 15	76	Relapse	TC, **SE**	+	+	0
MZL 16	47	Indolent	**SE**	+	−	TNFAIP3
MZL 17	28	Indolent	DXC, **SE**	+	−	0
MZL 18	18	Refractory	**SE**, RT	+	−	FBXW7
MZL 19	26	Relapse	DXC, **SE**	+	−	0
MZL 20	62	Relapse	TC, **SE**	+	−	0
SMLPD 1	3	Indolent	TC, **SE**, AF	−	+	TET2
SMLPD 2	54	Indolent	**SE**	−	+	0
SMLPD 3	16	Indolent	**SE**	−	+	0
SMLPD 4	38	Indolent	**SE**, DXC	+	+	0
SMLPD 5	68	Indolent	**SE**	−	+	0
SMLPD 6	1	Indolent	None	−	+	NA
SMLPD 7	22	Persistence	TC	−	+	0
SMLPD 8	61	Indolent	**SE**	+	+	0
SMLPD 9	0	Indolent	DXC, **SE**	−	+	0
SMLPD 10	36	Persistence	DXC, TC	−	+	0
SMLPD 11	7	Indolent	**SE**	−	+	0
SMLPD 12	32	Indolent	**SE**	−	+	0
SMLPD 13	33	Indolent	**SE**	+	+	0
PCTFHL 1	5	Refractory	RT, CT (GEMOX)	−	+	ARID1A
PCTFHL 2	50	Relapse	TC	−	+	0
PCTFHL 3	16	Refractory	CT (MTX)	−	+	SOCS1
PCTFHL 4	5	Lost to follow-up	NN	−	+	0
PCTFHL 5	25	Indolent	TC	−	+	0
cAITL 1	1	Systemic lymphoma (cAITL)	CT	−	+	TET2, TET2
cAITL 2	28		CT	Minor	+	0
cAITL 3	13		CT	−	+	0
cAITL 4	23		CT	−	Undet	0
cAITL 5	1		CT	Minor	+	TET2, TET2, RHOA
cAITL 6	4		CT	−	Undet	TET2, TET2, RHOA
cAITL 7	19		CT	−	Undet	NOTCH1, TET2
cAITL 8	3		CT	−	+	0
cAITL 9	19		CT	−	−	TET2, TET2
cAITL 10	5		CT	−	+	TET2, TET2, RHOA
cAITL 11	14		**SE**	−	+	TET2, TET2, RHOA

AF: antifungal cream, DLBCL: diffuse large B cell lymphoma, cAITL: cutaneous localization of angio-immunoblastic T lymphoma, CT: chemotherapy, DXC: doxycycline, RT: radiotherapy, MTX, methotrexate, MZL: (cutaneous) marginal zone lymphoma, NA: not available/analyzed, R: rituximab, SE: surgical excision, SMLPD: (Primary cutaneous CD4 +) small/medium T-cell lymphoproliferative disorder, TC: topical corticosteroids, PCTFHL: primary cutaneous T-follicular helper derived lymphoma, UNDET: undetermined.

**Table 4:** Comparison of clonality study and NGS data in AITL cases with both skin and nodal available biospies.

	Skin biopsy						Lymph node biopsy						Same monoclonal T population
	Monoclonal T-cell population	Gene	Exon	c.DNA	Protein	Type	Monoclonal T-cell population	Gene	Exon	c.DNA	Protein	Type	
cAITL 3	+	No mutation					+	No mutation					Yes
cAITL 7	+	TET2	8	c.3979C>T	p.(Gln1327*)	Nonsense	+	TET2	8	c.3979C>T	p.Gln1327*	Nonsense	Yes
		NOTCH1	28	c.5299C>T	p.(Leu1767Phe)	Missense		TET2	5	c.3577T>G	p.Cys1193Gly	Missense	
								RHOA	2	c.50G>T	p.Gly17Val	Missense	
cAITL 9	UNDET	TET2	6	c.3782G>A	p.Arg1261His	Missense	+	TET2	6	c.3782G>A	p.(Arg1261His)	Missense	No
		TET2	11	c.4579C>T	p.Gln1527*	Nonsense		TET2	11	c.4579C>T	p.(Gln1527*)	Nonsense	
cAITL 10	+	TET2	3	c.2383del	p.(Ser795Alafs*18)	Frameshift	+	TET2	3	c.2383delA	p.Ser795fs	Frameshift	Yes
		TET2	3	c.2593del	p.(Met865Cysfs*8)	Frameshift		TET2	3	c.2593delA	p.Met865fs	Frameshift	
		RHOA	2	c.50G>T	p.(Gly17Val)	Missense		RHOA	2	c.50G>T	p.Gly17Val	Missense	
								IDH2	4	c.515G>C	p.Arg172Thr	Missense	

C.DNA: coding DNA; cAITL: cutaneous localization of angio-immunoblastic T lymphoma; UNDET: T-cell clonality of undetermined significance.

The TNGS analysis (Table 3) was performed in all cases except for 1 MZL and 1 SMLPD due to a poor DNA quality. Pathogenic variants were found in 9 MZL (47%), 1 SMLPD (8%), 2 PCTFHL (40%) and 7 cAITL (64%). Pathogenic variants found in *MZL* were: *TNFAIP3* (6/19, 32%), *EP300* (4/19, 21%), *NOTCH2* (3/19, 16%), *KMT2D* (3/19, 16%), *CARD11* (2/19, 10.5%), *PLCG2* (1/19, 5%), *CREBBP* (1/19, 5%), *ITPKB* (1/19, 5%), *CIITA* (1/19, 5%), *GNA13* (1/19, 5%), and *FBXW7* (1/19, 5%). Despite good quality NGS data, few mutations were identified in SMLPD and PCTFHL. Pathogenic variant found in *SMLPD* was *TET2* (1/12, 8.5%). Pathogenic variants found in *PCTFHL* were *SOCS1* (1/5, 20%) and *ARID1A* (1/5, 20%). Patient with the *SOCS1* variant presented the following characteristics: presentation as multiple erythematous and infiltrated plaques without Sezary cells or circulating population, lichenoid pattern with epidermotropism, 60% of T-cells (CD7 lost) and 40% of B-cells, high expression of TFH markers (PD1 80%, CXCL13 50%, BCL6 20% CD10 10%). Patient with the *ARID1A* variant presented the following characteristics: multiple papules without Sezary cells or circulating population, lichenoid pattern separated from the epidermis by a grenz-zone and without epidermotropism, 80% of T-cells (CD7 lost) and 20% of B-cells, variable expression of TFH markers (PD1 95%, CXCL13 20%, BCL6 10%, no CD10 expression). Pathogenic variants found in *cAITL* were: *TET2* (n = 7, 64%), *RHOA* (n = 4, 36%; 3 *RHOA*^*G17V*^ and 1 *RHOA*^*G17L*^), and *NOTCH1* (1/11, 9%). Five cAITL displayed at least two pathogenic variant (*TET2* ± *RHOA* or *NOTCH1*), and two displayed only variants of *TET2*. Patients with *TET2* mutations had two *TET2* Single Nucleotide Variant (SNV) except one, with a single *TET2* SNV.

Molecular analysis had also been performed in addition to the skin tissue in lymph node biopsies of 4 AITL (Table 4). Cases no 3, 7 and 10 presented a similar monoclonal T population both samples, case no 9 had had a TCR gamma chain rearrangement with an undetermined ratio in the skin biopsy, and a true T monoclonal population in the lymph node. Cases no 7, 9 and 10, presented the same pathogenic variants in both samples, including *TET2* and/or *RHOA*^*G17V*^ hotspot.

## Discussion

The differential diagnosis of cutaneous lymphomas with TFH expression and/or hyperplasia is frequently a diagnostic challenge in daily diagnosis work. The spectrum of cutaneous T-cell lymphomas arising from TFH-cells extends from SMLPD to AITL^6^. Hyperplasia of TFH reactive T-cells is also frequently observe in cutaneous MZL^25^. The objective of the present study was to better characterize these different lesions (20 MZL, 13 SMLPD, 5 PCTFHL, and 11 cAITL) at the clinicopathological and molecular levels and to highlight tools to differentiate them.

The clinical presentation and histological appearance of the 49 cases described in this study were consistent with the literature^3,6,18,26^. In particular, MZL corresponded to erythematous papules/nodules (80%), or plaques (20%), which were most frequently located on the limbs (50%) and trunk (40%)^26^. Infiltrates in SMLPD could be separated into two patterns, as described by Beltzung et al.; (i) "Pattern 1" (n = 9/13, 69%): erythematous nodules, nodular/diffuse architecture, located on the head and neck (n = 4/9, 44%), followed by the trunk (n = 3/9, 33%) and upper extremities (n = 2/9, 23%); and (ii) "Pattern 2" (n = 4/13, 31%): erythematous-squamous plaques, lichenoid architecture, all located on the trunk^18^. Interestingly, loss of CD7 is described in the literature in 24% of SMLPD^18^. However, in this serie, none of the SMLPD cases showed CD7 loss, even partially. Although these data need to be confirmed in a larger cohort, it could be a tool to differentiate more aggressive forms of SMLPD, or even PCTFHL. Clinical presentation of PCTFHL was similar as described (multiple papules, plaques, and nodules of trunk/head)^5,6^, excepted that only one case presented a circulating monoclonal population (vs. 4/5 for Battistella et al.)^6^. Maculo-papular rash seems to be the classical presentation of cutaneous locations of AITL^3^.

MZL appears to differ clinically from SMLPD in their presentation as multiple skin nodules (p < 0.001). Histologically, nodular architecture (p < 0.01), presence of an FDC network (p < 0.001), and presence of monotypic plasma cells (p < 0.001) are characteristics that should favor the diagnosis of MZL rather than SMLPD. PD1 and CXCL13 were expressed in all SMLPD, but inconstantly in MZL (90% and 75% respectively), and with fewer positive cells in MZL than SMLPD: PD1 (19% in MZL vs. 49% in SMLPD, p = 0.016), CXCL13 (8% in MZL vs. 20% in SMLPD, p = 0.03), or BCL6 (2.5% in MZL vs. 11% in SMLPD, p = 0.27). PD1 expression profile was also different in MZL and SMLPD ; PD1 was expressed on medium to large and frequently clustered cells in SMLPD^18^, whereas, in MZL, PD1 was expressed by the small T-cells of the germinal centers.

Concerning T-cell lymphoproliferations/lymphomas, features in favor of a more aggressive disease such as PCTFHL or AITL are: elderly patients (p < 0.01), presentation as multiple (p < 0.001) erythematous and scaly patches or as a maculopapular rash for cAITL (p < 0.001), association with systemic symptoms and/or biological alterations (p < 0.001), interstitial architecture (p < 0.001), loss of T-cell markers (p < 0.001), and an elevated proliferative index (p < 0.01). Concerning TFH markers, there was no difference in the amount of stained cells with PD1 (p = 0.12) or CXCL13 (p = 0.79) across these subtypes, but BCL6 (p = 0.024) and CD10 (p = 0.08) seemed more often positive in cAITL and PCTFHL. Extension assessment, including a CT scan and immunophenotyping of the circulating blood, is particularly important to differentiate PCTFHL from cAITL.

In this study, the main objective was to compare these entities. That’s why it was decided to include only cases with a monoclonal population, to try to include only "typical" cases and to limit the possibility of including inflammatory diseases, as, in the skin, dominant clones may also be found in a variety of benign dermatoses. Literature has already proved the interest of clonality assessment in the diagnosis of cutaneous lymphomas, the finding of a monoclonal population orienting the diagnosis in more than 80% of MZL^26^, more than 65% of SMLPD^18,27^, and all PCTFHL^6^. This study further emphasizes the importance of an integrated histomolecular diagnosis. Indeed, 4 MZL presented a PD1 expression with more than 30% of stained cells, making the differential diagnosis with SMLPD particularly difficult without molecular data. In these patients, a monoclonal B-cell population was found, without monoclonal T-cell population, and the diagnosis of MZL could be made. The study of clonality may therefore be helpful in these cases of MZL with TFH hyperplasia of the microenvironnement^10^. Moreover, this study also highlights the value of comparing molecular techniques in cases of suspected cutaneous localization of systemic lymphoma. The discovery of the same monoclonal population (AITL no 3) or same NGS pathogenic variants (AITL no 7, 9 and 10), in skin biopsy and lymph node can, indeed, allow to link two locations of the same disease.

Taken individually, TNGS also identified pathogenic variants helpful for the diagnosis in 42% of MZL and 64% of cAITL. Even if these cases do not represent a large majority, they are not negligible considering the importance of an optimal classification of these lesions for the management and prognosis.

In the present study, 47% of MZL presented pathogenic variants, involving *TNFAIP3* (n = 6, 32%), *KMT2D* (n = 3, 16%), *EP300* (n = 4, 21%), *NOTCH2* (n = 3, 16%), and *CARD11* (n = 2, 10.5%). According to the Cosmic database, *NOTCH2* (19%), *KMT2D* (15%), and *TNFAIP3* (11%) are the top 3 most frequent mutated genes observed in mucosa-associated lymphoid tissue (MALT) lymphomas^28^ and nodal MZL^29^. *EP300* and *CARD11* are also frequently described in MZL^30^. *FBW7* has not been considered as helpful^31^. Mutations do not seem correlated to prognosis in MZL: 44% of indolent cases among the mutated cases and 60% of relapses among the non-mutated cases. However, the panel targeted the most relevant genes for lymphoma diagnosis but did not cover all exons for some genes (e.g., *KMT2D*) and lacked a few select genes (e.g., *FAS, SLAMF1, SPEN,* and *NCOR2*), which are described in cutaneous MZL^10,32^. This could explain the negativity of some cases and the lack of correlation with the clinical course.

Concerning AITL, most studies concern nodal locations, in which mutations of *TET2* (52–76%), *IDH2* (20–45%), *DNMT3A* (30–40%) and *RHOA G17V* (28–70%) seem frequent^11–16^. The only study currently published in cutaneous localizations is the one of Leclaire Alirkilicarslan et al., which included 41 patients and found *IDH2 R172K/S* and *RHOA G17V* mutations in 19% and 78% of cases respectively using PCR^17^. This study is the only one to study mutations using TNGS on a cohort of cutaneous localizations of AITL. Among them, 64% presented pathogenic variants involving *TET2* (n = 7, 64%), *RHOA* (n = 4, 36%), and *NOTCH1* (n = 1, 9%). Leclaire Alirkilicarslan et al. found 78% of *RHOA* G17V and 19% of cases with *IDH2* R172 substitutions using PCR in a cohort of cAITL. In nodal AITL, mutations are much more frequent^11,12,15^; *TET2* SNV (76%), *RHOA* G17V (60–78%), and *IDH2* R172 substitutions (19.5%). In the present study, the percentage of mutated cases was lower than described in the literature, and no *IDH2* pathogenic variants were found. This may be explained by the small number of cases in the study or by the low density of tumoral cells in the samples. However, these molecular findings were confirmative of the diagnosis in 64% of cases, as in 4/5 cases with an atypical presentation (slight perivascular infiltrates, no identifiable atypical lymphocytes) in the study of Leclaire Alirkilicarslan et al. These data suggest that TNGS may represent a useful tool for the diagnosis of cAITL. The VAF of the SNV were lower in cAITL (average = 6.7%, median = 3.1%, ranging from 1.2 to 22%), compared to 14.2% in MZL and 45.2% in PCTFHL. It can be explained by the low cell density in these biopsies (less than 25% of the surface) or by clonal tumor heterogeneity, as already described in these entities^13,33^. Nevertheless, the sequencing quality remains optimal; the described variants are robust due to a minimal depth of 700× (Suppl. Table 3).

The second objective of this study was to decipher the spectrum of TFH lymphomas, including also lymphoproliferations and controversial intermediate forms. Concerning SMLPD, only one case (8%) presented a pathogenic variant of *TET2,* which may correspond to clonal hematopoiesis. Beltzung et al. reported a unique pathogenic variant of *DNMT3A* among 13 SMLPD, but this gene was not part of our panel^18^. Concerning PCTFHL, two cases (40%) presented isolated pathogenic variants of *ARID1A* and *SOCS1*. These two mutated cases required aggressive treatment, whereas the two cases with an indolent disease did not present any detectable variant. *ARID1A* mutations are reported in numerous cancers and lymphomas and therefore does not seem really helpful in isolation^15,34^. *SOCS1* was a class 3 of uncertain clinical significance^35,36^. This variant has already been described in TFH lymphomas^34^, but also in MF^37^. This finding was arguable, PCTFHL currently not being a recognized entity, and of difficult differential diagnosis with MF expressing TFH markers or Sezary syndrome^38,39^. In PCTFHL no 3 in particular, the clinical presentation as erythematosquamous plaques, the presence of epidermotropism, and the *SOCS1* mutation may have suggested the diagnosis of MF or Sezary syndrome. Nevertheless, the absence of Sezary cells in the blood, the presence of interface dermatitis lesions, the polymorphism of the infiltrate (presence of 50% of B-cells and numerous plasma cells), and the intense expression of all TFH markers (PD1: 80%, CXCL13: 50%, BCL6: 20%, CD10: 10%) pleaded against this diagnosis. Within the panel studied, there does not appear to be a common or recurrent molecular profile between SMLPD, PCTFHL, or cAITL. The molecular abnormalities usually present in AITL were not found in SMLPD or PCTFHL. These data remain debatable and future studies will undoubtedly allow a better classification of these case. Indeed, one of the raised hypotheses was that the spectrum of TFH-lesions was potentially underpinned by a mutational spectrum. The frequency of mutations found in TNGS (8% in SMLPD, 40% in PCTFHL and 64% in cAITL) seems to go in the same direction as the prognosis of these lesions. As already described in nodal AITL, these data may suggest that *RHOA* mutation may be a secondary event in lymphomatous cells after other critical molecular events such as *TET2* mutations that first occur in "premalignant" lymphocyte precursors^13,17,40–42^. This hypothesis may agrees with the distribution of pathogenic variants in the present study and reinforces the theory of a spectrum of lymphoma derived from a TFH lymphocyte. These data will need to be confirmed in a larger cohort, gathering a larger number of PCTFHL cases. If accepted, the provisional PCTFHL entity will also need to be defined, in particular its exact terminology (lymphoproliferation or lymphoma), and diagnosis criteria. The controversial nature of these intermediate forms makes it difficult to include these patients in studies.

This study highlights some histological features to distinguish the main subtypes of cutaneous lymphomas expressing TFH markers, that might reasonably be considered in a differential diagnosis. It also underlines the interest of integrated histomolecular diagnosis, using clonality and NGS, to classify these pathologies and strength the hypothesis of a spectrum of cutaneous lymphomas arising from a T-follicular helper lymphocyte is, even if further studies on a more significant number of patients are required to draw firm conclusions. In particular, the provisional entity so-called PCTFHL will need to be defined thanks to larger cohorts, from clinical, histological, and molecular points of view, in order to determine if it related to lymphoproliferations (such as SMLPD), or to real lymphomas with TFH phenotype, by analogy to nodal lymphomas.

## Supplementary Information

Supplementary Tables.

Supplementary Information.

## Data Availability

All data generated or analyzed during this study are included in this published article (and its supplementary information files).
