# Supplementary material for: Deciphering the spectrum of cutaneous lymphomas expressing TFH markers
Source: Sci Rep. 2023 Apr 20;13:6500. doi: 10.1038/s41598-023-33031-3 (PMC10119163; doi:10.1038/s41598-023-33031-3)
Supplement: Supplementary file 1 — Supplementary Tables. [file 41598_2023_33031_MOESM1_ESM.docx]

**Supplemental Table S1**: **References of antibodies used for immunohistochemistry.**

| Antibodies/*In situ* hybridization | References |
| --- | --- |
| Anti-CD20 | Mouse monoclonal primary antibody, L26 ; Agilent Dako, Boston, USA |
| Anti-CD3 | Rabbit polyclonal primary antibody, A0452 ; Agilent Dako, Boston, USA |
| Anti-CD2 | Mouse monoclonal primary antibody, MRQ11 ; Roche Diagnostics; Meylan; France |
| Anti-CD5 | Rabbit monoclonal primary antibody, SP 19 ; Roche Diagnostics; Meylan; France |
| Anti-CD4 | Rabbit monoclonal primary antibody, SP 57 ; Roche Diagnostics; Meylan; France |
| Anti-CD8 | Rabbit monoclonal primary antibody, SP 57 ; Roche Diagnostics; Meylan; France |
| Anti-CD7 | Rabbit monoclonal primary antibody, SP 94 ; Roche Diagnostics; Meylan; France |
| Anti-CD138 | Mouse monoclonal primary antibody, B-A38 ; Roche Diagnostics; Meylan; France |
| Anti-Kappa | Rabbit polyclonal primary antibody, A0191; Agilent Dako, Boston, USA |
| Anti-Lambda | Rabbit polyclonal primary antibody, A0193 ; Agilent Dako, Boston, USA |
| Anti-PD1 | Mouse monoclonal primary antibody, NAT105 ; Roche Diagnostics; Meylan, France |
| Anti-ICOS | Rabbit monoclonal primary antibody, AB105227 ; Abcam, Cambridge, UK |
| Anti-CXCL13 | Goat polyclonal primary antibody, BCA-1 ; R&D System, Minneapolis, USA |
| Anti-CD10 | Rabbit monoclonal primary antibody, SP67; Roche Diagnostics; Meylan; France |
| Anti-BCL6 | Mouse monoclonal primary antibody, GI191E/A8 ; Roche Diagnostics; Meylan; France |
| Anti-CD23 | Rabbit monoclonal primary antibody, SP23 ; Thermofisher, Waltham, US |
| Anti-CD21 | Rabbit monoclonal primary antibody, 2G9 ; Roche Diagnostics; Meylan; France |
| Anti-CD30 | Mouse monoclonal primary antibody, M0751 ; Agilent Dako, Boston, USA |
| Ki67/MIB1 | Mouse monoclonal primary antibody, M7240; Agilent Dako, Boston, USA |
| HIS EBV encoded RNAs | 800-2842; Roche Diagnostics, Ventana; Medical Systems; Mannheim; Allemagne |

**Supplemental Table S2: NGS panel focusing on 47 genes used in the present study.**

| Genes of the NGS Panel | Targeted exons | Transcripts |
| --- | --- | --- |
| ARID1A | 1-5, 8, 12-15, 17-20 | NM_006015 |
| BCL2 | 2a | NM_000633 |
| BCOR | 4, 10 | NM_001123385 |
| BIRC3 | 3 à 9 | NM_001165.4 |
| BRAF | 15 | NM_004333 |
| BTK | 14-16 | NM_000061_2 |
| CARD11 | 4 à 9 | NM_032415.5 |
| CCND1 | 1-5 | NM_053056 |
| CD58 | 2, 3 | NM_001779.2 |
| CD79A | 2-5 | NM_001783 |
| CD79b | 2-6 | NM_000626.3 |
| CDKN2A | full cds (1 à 3) + utr (CNV) | NM_000077 |
| CDKN2B | 1,2 + utr CNV | NM_004936 |
| CIITA | 9, 11, 16 | NM_000246.3 |
| CREBBP | 2, 24 à 30 | NM_004380 |
| CXCR4 | 1, 2 | NM_003467 |
| EP300 | 1-3, 5, 6, 9, 10, 14, 15, 18, 20, 25-31 | NM_001429.3 |
| EZH2 | 12, 16-18 | NM_004456.4 |
| FBXW7 | 9-11 | NM_033632.3 |
| FOXO1 | 1, 2 | NM_002015.3 |
| GNA13 | 1-4 | NM_006572.5 |
| ID3 | 1, 2 | NM_002167.4 |
| IDH2 | 4 | NM_002168.3 |
| IRF4 | 2, 3 | NM_001195286.1 |
| ITPKB | 2 | NM_002221.3 |
| KLF2 | 2, 3 | NM_016270.2 |
| KMT2D/MLL2 | 5, 6, 8, 10,11, 13-17, 20-23, 31-34, 36, 38-40, 42, 44, 46-51, 53 | NM_003482 |
| MEF2B | 3 | NM_001145785.1 |
| MFHAS1 | 1 | NM_004225.2 |
| MYD88 | 2-5 | NM_001172567.1 |
| NOTCH1 | 26-28, 34 | NM_017617.3 |
| NOTCH2 | 26-28, 34 | NM_024408.2 |
| PIM1 | 1-6 | NM_001243186.1 |
| PLCG2 | 2, 10, 13, 14, 18, 28-30 | NM_002661 |
| PRDM1 | 2-7 | NM_001198.3 |
| PTPRD | 21, 42 à 48 | NM_002839 |
| RHOA | 2-5 | NM_001313941.1 |
| SF3B1 | 14-18 | NM_012433.2 |
| SOCS1 | 2 | NM_003745.1 |
| STAT3 | 21b | NM_139276.2 |
| STAT6 | 4, 5 | NM_001178078 |
| TCF3 | 1, 9, 15,16, 18 | NM_001136139 |
| TET2 | 3-11 | NM_001127208.2 |
| TNFAIP3 | 2-4, 6-9 | NM_001270508.1 |
| TRAF2 | 2, 4 | NM_021138 |
| TRAF3 | 5, 10 | NM_145725 |
| XPO1 | 15-18 | NM_003400 |

**Supplemental Table S3: Catalogue of mutations displayed by the mutated cases of the study**; Depth of coverage systematically of at least 35X (routinely recommended), and furthermore 50X (due to the use of paraffin embedded samples). Only class 3 to 5 variants according to the Cosmic database were retained (class 5 : pathogenic, class 4 : likely pathogenic, and class 3 : variant of unknown significance).

MZL: marginal zone lymphomas, SMLPD: Primary cutaneous CD4‐positive small/medium T‐cell lymphoproliferative disorder, PCTFHL : primary cutaneous T-follicular helper derived lymphoma, cAITL : angioimmunoblastic T cell lymphoma.

|  | gene | Transcrit | Exon | c.DNA | protein | type of mutation | depth | VF,% | class |
| --- | --- | --- | --- | --- | --- | --- | --- | --- | --- |
| MZL 1 | CARD11 | NM_032415 | 9 | c.1234G>A | p.(Glu412Lys) | missense | 2884 | 2,6 | 4 |
|  | CARD11 | NM_032415 | 6 | c.692A>C | p.(Gln231Pro) | missense | 2311 | 5 | 4 |
|  | EP300 | NM_001429 | 31 | c.5368T>A | p.(Cys1790Ser) | missense | 3854 | 14,2 | 4 |
|  | TNFAIP3 | NM_001270507 | 8 | c.2036T>C | p.(Ile679Thr) | missense | 3020 | 42,9 | 4 |
| MZL 2 | ITPKB | NM_002221 | 2 | c.178C>G | p.Pro60Ala | missense | 778 | 20,00 | 3 |
|  | ITPKB | NM_002221 | 2 | c.199G>T | p.Glu67* | nonsense | 729 | 7,40 | 4 |
|  | KMT2D | NM_003482 | 47 | c.14536delG | p.Glu4846Lysfs*12 | frameshift | 722 | 9,50 | 5 |
|  | NOTCH2 | NM_024408 | 28 | c.5120delG | p.Gly1707Alafs*31 | frameshift | 898 | 3,20 | 4 |
|  | TNFAIP3 | NM_001270507 | 7 | c.1681C>T | p.Gln561* | nonsense | 955 | 9,40 | 5 |
| MZL 5 | CARD11 | NM_032415 | 7 | c.1010G>A | p.(Arg337Gln) | missense | 4332 | 28,50 | 4 |
|  | KMT2D | NM_003482 | 21 | c.5104C>T | p.(Arg1702*) | nonsense | 4062 | 26,20 | 5 |
|  | NOTCH2 | NM_024408 | 34 | c.6379_6399delinsT | p.(Lys2127Phefs*4) | frameshift | 4212 | 22,00 | 5 |
|  | TNFAIP3 | NM_001270507 | 4 | c.577_580del | p.(Glu193Tyrfs*22) | frameshift | 3113 | 38,50 | 5 |
| MZL 8 | TNFAIP3 | NM_001270507 | 9 | c.2090G>A | p.(Arg697Lys) | missense | 4439 | 48,20 | 3 |
|  | EP300 | NM_001429 | 29 | c.4751T>G | p.(Leu1584Arg) | missense | 3558 | 4,30 | 4 |
|  | PLCG2 | NM_002661 | 28 | c.3101C>T | p.(Thr1034Met) | missense | 4095 | 47,00 | 3 |
| MZL 10 | CREBBP | NM_001079846 | 29 | c.4925_4927delCCT | p.Ser1642del | inframe_3 | 6723 | 3,10 | 5 |
|  | EP300 | NM_001429 | 27 | c.4373C>G | p.Pro1458Arg | missense | 9624 | 3,10 | 4 |
| MZL 11 | KMT2D | NM_003482 | 34 | c.9625delC | p.Leu3209Cysfs*12 | frameshift | 5873 | 7,4 | 5 |
| MZL 13 | CIITA | NM_000246 | 11 | c.1907C>A | p.(Thr636Lys) | missense | 5928 | 4,4 | 3 |
|  | EP300 | NM_001429 | 27 | c.4399T>C | p.(Tyr1467His) | missense | 4377 | 3,8 | 4 |
|  | GNA13 | NM_006572 | 1 | c.31C>G | p.(Leu11Val) | missense | 5167 | 3,5 | 4 |
|  | NOTCH2 | NM_024408 | 34 | c.6439A>G | p.(Thr2147Ala) | missense | 4655 | 3,5 | 4 |
|  | TNFAIP3 | NM_001270507 | 8 | c.1939A>C | p.(Thr647Pro) | missense | 4015 | 46,1 | 3 |
|  | TNFAIP3 | NM_001270507 | 4 | c.504G>A | p.(Trp168*) | nonsense | 4485 | 2,7 | 5 |
| MZL 16 | TNFAIP3 | NM_001270507 | 8 | c.2000del | p.(Ser667Thrfs*30) | frameshift | 2913 | 13,30 | 5 |
| MZL 18 | FBXW7 | NM_001013415 | 8 | c.1040G>A | p.(Arg347His) | missense | 1649 | 2,20 | 4 |
| SMLPD 1 | TET2 | NM_017628 | 3 | c.2578C>T | p.Gln860* | nonsense | 7228 | 6,2 | 3 |
| SMLPD 13 | DNMT3A |  | 20 | c.2374dupC | p.Arg792fs | frameshift_variant | 12429 | 17 | 5 |
| PCTFHL 1 | ARID1A | NM_006015 | 18 | c.4372C>T | p.Gln1458* | nonsense | 928 | 70,2 | 5 |
| PCTFHL 3 | SOCS1 |  | 2 | c.218_222dupTCCTG | p.Asp75Serfs*12 | frameshift | 3003 | 20,2 | 3 |
| AITL 1 | TET2 | NM_001127208 | 6 | c.3782G>A | p.(Arg1261His) | missense | 5137 | 9,6 | 5 |
|  | TET2 | NM_001127208 | 11 | c.5582G>A | p.(Gly1861Glu) | missense | 5346 | 21,9 | 4 |
| AITL 5 | RHOA | NM_001664 | 2 | c.50G>T | p.(Gly17Val) | missense | 4967 | 14,1 | 5 |
|  | TET2 | NM_017628 | 3 | c.2466del | p.(Met823*) | frameshift | 5336 | 15 | 5 |
|  | TET2 | NM_001127208 | 11 | c.5500C>T | p.(Gln1834*) | nonsense | 5683 | 17,1 | 4 |
| AITL 6 | TET2 | NM_001127208 | 6 | c.3764dupA | p.(Tyr1255*) | nonsense | 6410 | 3,9 | 5 |
|  | TET2 | NM_001127208 | 11 | c.4661_4664del | p.(Thr1554Serfs*16) | frameshift | 4221 | 12,6 | 5 |
|  | RHOA | NM_001664 | 2 | c.50G>T | p.(Gly17Val) | missense | 6932 | 3,1 | 4 |
| AITL 7 | TET2 | NM_001127208 | 8 | c.3979C>T | p.(Gln1327*) | nonsense | 4262 | 1,2 | 5 |
|  | NOTCH1 | NM_017617 | 28 | c.5299C>T | p.(Leu1767Phe) | missense | 2497 | 4,2 | 4 |
| AITL 9 | TET2 | NM_001127208 | 6 | c.3782G>A | p.Arg1261His | missense | 12779 | 2,9 | 4 |
|  | TET2 | NM_001127208 | 11 | c.4579C>T | p.Gln1527* | nonsense | 5535 | 3,1 | 4 |
| AITL 10 | TET2 | NM_017628 | 3 | c.2383del | p.(Ser795Alafs*18) | frameshift | 5666 | 1,5 | 5 |
|  | TET2 | NM_017628 | 3 | c.2593del | p.(Met865Cysfs*8) | frameshift | 5382 | 1,9 | 5 |
|  | RHOA | NM_001664 | 2 | c.50G>T | p.(Gly17Val) | missense | 5289 | 2,8 | 4 |
| AITL 11 | RHOA | NM_001664 | 3 | c.49_50delGGinsTT | p.Gly17Leu | missense | 3547 | 2,4 | 5 |
|  | TET2 | NM_001127208 | 5 | c.3546T>G | p.Tyr1182* | stop_gained | 4220 | 1,5 | 5 |
|  | TET2 | NM_001127208 | 7 | c.3811dupT | p.Cys1271Leufs*29 | frameshift | 4927 | 2 | 5 |

**Supplemental Table S4: Library Quality Data Report**

Library Quality Data Report in all cases except for 1 MZL and 1 SMLPD, for which the NGS technique could not be performed (NA: not analysed) due to insufficient amount of DNA.

MZL: marginal zone lymphomas, SMLPD: Primary cutaneous CD4‐positive small/medium T‐cell lymphoproliferative disorder, PCTFHL : primary cutaneous T-follicular helper derived lymphoma, cAITL : angioimmunoblastic T cell lymphoma.

| Case | Number of reads | Percentage of mapped reads | Percentage of mapped bp’s on-target | Percentage of target regions with 200X coverage | Percentage of target regions with 500X coverage | Percentage of target regions with 1000X coverage | coverage 10% quantile | coverage heterogeneity |
| --- | --- | --- | --- | --- | --- | --- | --- | --- |
| MZL 1 | 4377510 | 98,33% | 71,18% | 100,00% | 99,91% | 99,00% | 1722x | 0,25% |
| MZL 2 | 849308 | 96,92% | 72,65% | 98,53% | 74,72% | 13,20% | 380x | 0,39% |
| MZL 3 | 14 752 728 | 96,06% | 53,19% | 100,00% | 100,00% | 100,00% | 4826x | 0,62% |
| MZL 4 | NA | NA | NA | NA | NA | NA | NA | NA |
| MZL 5 | 7074658 | 96,24% | 53,29% | 100,00% | 100,00% | 99,80% | 3305x | 0,12% |
| MZL 6 | 9 381 170 | 97,42% | 72,82% | 100,00% | 100% | 100,00% | 5284x | 0,00% |
| MZL 7 | 6 161 654 | 98,09% | 73,58% | 100,00% | 100% | 100,00% | 3817x | 0,00% |
| MZL 8 | 6294150 | 96,16% | 50,14% | 100,00% | 99,89% | 99,70% | 2688x | 0,22% |
| MZL 9 | 3 725 110 | 98,33% | 51,53% | 100,00% | 99,71% | 98,35% | 144x | 0,17% |
| MZL 10 | 11064804 | 96,64% | 78,10% | 100,00% | 100,00% | 100,00% | 7500x | 0,00% |
| MZL 11 | 7761532 | 98,18% | 78,53% | 100,00% | 100,00% | 100,00% | 4129x | 0,02% |
| MZL 12 | 8 409 386 | 96,06% | 50,84% | 100,00% | 100,00% | 99,81% | 3954x | 0,19% |
| MZL 13 | 16792896 | 97,33% | 65,88% | 100,00% | 100,00% | 100,00% | 8804x | 0,18% |
| MZL 14 | 4 833 108 | 98,18% | 78,20% | 100,00% | 100,00% | 99,98% | 2977x | 0,00% |
| MZL 15 | 9 308 954 | 95,89% | 52,50% | 100,00% | 100,00% | 99,86% | 4467x | 0,17% |
| MZL 16 | 4837682 | 98,29% | 51,45% | 100,00% | 99,79% | 99,20% | 1837x | 0,23% |
| MZL 18 | 4168272 | 98,28% | 54,85% | 100,00% | 99,95% | 99,50% | 1778x | 0,03% |
| MZL 19 | 9467894 | 97,67% | 74,65% | 100,00% | 100,00% | 100,00% | 6659x | 0,00% |
| MZL 20 | 7 457 784 | 97,80% | 53,29% | 100,00% | 99,97% | 99,73% | 3241x | 0,23% |
| SMLPD 1 | 5182974 | 98,01% | 70,20% | 100,00% | 100,00% | 99,30% | 1807x | 0,05% |
| SMLPD 2 | 6307012 | 97,99% | 74,77% | 100,00% | 100,00% | 100,00% | 3906x | 0,00% |
| SMLPD 3 | 6 348 342 | 98,06% | 77,05% | 100,00% | 100,00% | 100,00% | 3432x | 0,00% |
| SMLPD 4 | 8669432 | 96,77% | 57,15% | 100,00% | 100,00% | 100,00% | 4340x | 0,00% |
| SMLPD 5 | 7 992 000 | 98,04% | 67,79% | 100,00% | 100,00% | 99,81% | 4046x | 0,23% |
| SMLPD 6 | NA | NA | NA | NA | NA | NA | NA | NA |
| SMLPD 7 | 7 300 510 | 96,33% | 74,63% | 100,00% | 100,00% | 100,00% | 5037x | 0,00% |
| SMLPD 8 | 8954240 | 95,87% | 76,38% | 100,00% | 100,00% | 100,00% | 5456x | 0,00% |
| SMLPD 9 | 5 631 706 | 97,90% | 78,86% | 100,00% | 100,00% | 99,86% | 3325x | 0,12% |
| SMLPD 10 | 5 475 700 | 97,18% | 75,93% | 100,00% | 98,54% | 96,76% | 2232x | 3,36% |
| SMLPD 11 | 1391946 | 98,03% | 55,36% | 93,59% | 78,75% | 42,10% | 275x | 5,30% |
| SMLPD 12 | 883 148 | 95,26% | 84,24% | 99,48% | 86,27% | 25,57% | 461x | 0,16% |
| SMLPD 13 | 13732882 | 94,17% | 75,31% | 100,00% | 100,00% | 100,00% | 7302x | 0.13% |
| TFHL 1 | 2152096 | 96,91% | 74,34% | 100,00% | 98,90% | 90,70% | 1039x | 0,27% |
| TFHL 2 | 11 017 356 | 98,31% | 61,02% | 100,00% | 100,00% | 100,00% | 4822x | 0,01% |
| TFHL 3 | 4283537 | 99,53% | 69,35% | 100,00% | 100,00% | 98.9% | 1653x | 0.17% |
| TFHL 4 | 4 258 052 | 98,37% | 63,44% | 100,00% | 100,00% | 99,12% | 168x | 0,01% |
| TFHL 5 | 9286518 | 97,08 | 79,98 | 100.00% | 100.00% | 100.00% | 5974x | 0,01 |
| AITL 1 | 6 826 528 | 98,17% | 63,13% | 100,00% | 99,99% | 99,75% | 2918x | 0,27% |
| AITL 2 | 9286080 | 96,28% | 77,79% | 100,00% | 100,00% | 100,00% | 5891x | 0,02% |
| AITL 3 | 4768814 | 96,95% | 79,51% | 100,00% | 100,00% | 99,87% | 2977x | 0,07% |
| AITL 4 | 432856 | 93,10% | 79,58% | 93,96% | 11,37% | 0,10% | 223x | 0,00% |
| AITL 5 | 8473026 | 98,01% | 62,37% | 100,00% | 100,00% | 100,00% | 4156x | 0,00% |
| AITL 6 | 8434800 | 98,24% | 76,37% | 100,00% | 100,00% | 100,00% | 5131x | 0,06% |
| AITL 7 | 6375090 | 98,33% | 77,78% | 100,00% | 100,00% | 100,00% | 3717x | 0,07% |
| AITL 8 | 1 933 674 | 97,78% | 79,87% | 99,98% | 98,67% | 75,65% | 774x | 0,09% |
| AITL 9 | 9326616 | 97,16% | 77,86% | 100,00% | 100,00% | 100,00% | 5804x | 0,00% |
| AITL 10 | 5366684 | 97,86% | 80,22% | 100,00% | 100,00% | 100,00% | 3765x | 0,00% |
| AITL 11 | 9424416 | 96,64% | 77,44% | 100,00% | 100,00% | 100,00% | 6404x | 0,00% |
