# Supplementary material for: Deciphering the spectrum of cutaneous lymphomas expressing TFH markers
Source: Sci Rep. 2023 Apr 20;13:6500. doi: 10.1038/s41598-023-33031-3 (PMC10119163; doi:10.1038/s41598-023-33031-3)
Supplement: Supplementary file 2 — Supplementary Information. [file 41598_2023_33031_MOESM2_ESM.docx]

**Supplemental Materiel and methods :**

**Immunohistochemistry**

Three μm-thick tissue sections were cut from paraffin-embedded skin biopsy, dried, de-waxed, rehydrated and slides were unmasked with ER1 Solution (Leica, Bond Epitope Retrieval Solution 1). Detailed references of used antibodies are available in supplemental data (suppl. Table 1). Immunochemistry was performed using standard procedures in an automated Immunostainer (Leica BOND-MAX, LEICA MICROSYSTEMES SA, NANTERRE, France ). In situ hybridization for Epstein-Barr virus (EBV) was performed on deparaffinized tissue sections using a fluorescein isothiocyanatecoupled specific peptidic nucleic acid probe (Ventana BenchMark XT automated immunostainer).

**DNA extraction and libraries preparations**

DNA was extracted with a Qiacube device from formalin-fixed paraffin-embedded (FFPE) tissue samples according to the manufacturer’s instructions (QiAamp DNA mini kit, Qiagen, Courtaboeuf, France). DNA concentration was measured with a Qubit Fluorometer using a dsDNA HS assay (Thermo Fisher Scientific Inc, Courtaboeuf, France). Genomic DNAs were tested for TNGS using a custom design-panel based on a capture enrichment strategy (Sophia Genetics, Lausanne, Switzerland). Libraries were performed using a Kapa Hyper plus library kit (Roche NimbleGen Madison, Wisconsin, USA) from 50ng of genomic DNA. Libraries were pooled per 12 samples using 150ng of each one, dried using a vacuum DNA concentrator (SpeedVac, ThermoFisher Scientific Inc, Courtaœuf, France) until the mix was completely lyophilized. For the capture step, the pooled libraries were suspended in hybridization buffer and incubated with custom xGen® Lockdown Probes (Sophia Genetics) for 16 hours at 65°C. After washing steps using buffers of different stringency, captured DNA was amplified by PCR (15 cycles). The PCR products were cleaned, quantified (dsDNA HS Assay kit) and the quality as checked on DNA chips (Agilent). Each pool was diluted to 4nM before mixing pools in equal amounts and sequenced on a NextSeq500 sequencer using the NextSeq500 Mid Output Kit v2 (300 cycles) chemistry (Illumina, San Diego, CA, USA).
